# Prevalence of the use of cancer related self-tests by members of the public: a community survey

**DOI:** 10.1186/1471-2407-6-215

**Published:** 2006-08-25

**Authors:** Sue Wilson, Sheila Greenfield, Helen M Pattison, Angela Ryan, Richard J McManus, David Fitzmaurice, John Marriott, Cyril Chapman, Sue Clifford

**Affiliations:** 1 Department of Primary Care and General Practice, The University of Birmingham, Edgbaston, Birmingham, B15 2TT, UK; 2 School of Life and Health Sciences, Aston University, Aston Triangle, Birmingham, B4 7ET, UK; 3 Regional Genetics Service, The Women's Hospital, Birmingham, B15 2TJ, UK

## Abstract

**Background:**

Self-tests are those where an individual can obtain a result without recourse to a health professional, by getting a result immediately or by sending a sample to a laboratory that returns the result directly. Self-tests can be diagnostic, for disease monitoring, or both. There are currently tests for more than 20 different conditions available to the UK public, and self-testing is marketed as a way of alerting people to serious health problems so they can seek medical help. Almost nothing is known about the extent to which people self-test for cancer or why they do this. Self-tests for cancer could alter perceptions of risk and health behaviour, cause psychological morbidity and have a significant impact on the demand for healthcare. This study aims to gain an understanding of the frequency of self-testing for cancer and characteristics of users.

**Methods:**

Cross-sectional survey. Adults registered in participating general practices in the West Midlands Region, will be asked to complete a questionnaire that will collect socio-demographic information and basic data regarding previous and potential future use of self-test kits. The only exclusions will be people who the GP feels it would be inappropriate to send a questionnaire, for example because they are unable to give informed consent. Freepost envelopes will be included and non-responders will receive one reminder. Standardised prevalence rates will be estimated.

**Discussion:**

Cancer related self-tests, currently available from pharmacies or over the Internet, include faecal occult blood tests (related to bowel cancer), prostate specific antigen tests (related to prostate cancer), breast cancer kits (self examination guide) and haematuria tests (related to urinary tract cancers). The effect of an increase in self-testing for cancer is unknown but may be considerable: it may affect the delivery of population based screening programmes; empower patients or cause unnecessary anxiety; reduce costs on existing healthcare services or increase demand to investigate patients with positive test results. It is important that more is known about the characteristics of those who are using self-tests if we are to determine the potential impact on health services and the public.

## Background

Self-tests are those where an individual can obtain a result without recourse to a health professional: by getting a result immediately (e.g. most prostate specific antigen (PSA) and faecal occult blood (FOB) tests), or by sending a sample to a laboratory that returns the result directly to the individual (e.g. some chlamydia tests). Self-tests can be diagnostic (e.g. urine tests for pregnancy), for disease monitoring (e.g. blood pressure), or both (e.g. PSA tests). There are currently tests for more than 20 different conditions available to the UK public [1].

A number of reports have expressed concern about the development of self-testing [2,3]. Potential problems highlighted include: lack of professional support when receiving bad news; lack of expertise in interpreting results and action needed; unreliable results generating false security or anxiety; that individuals may be forced to take tests by people other than health professionals (e.g. employers); the potential break-down of public health surveillance; that commercially driven test development may lead to demands for further testing or treatment which the NHS is unable to meet. Much coverage of self-testing in the press is also negative, warning of the unreliability of tests and dangers of misunderstanding medical information [4-7]. However, such reports do not seem to have deterred users. Market research reports that "almost six in ten Britons diagnose themselves at home with self-testing equipment instead of going to the doctor", although this does include thermometers [8]. Sales of self-testing equipment are reported to have increased dramatically: almost £54.3 m was spent on self-diagnostic products in 2002, a 32% growth since 1998 [9], and it has been predicted that this will rise to over £60 m by 2007 [8]. Market surveys in the US indicate at least 25% of all medical tests are conducted outside the hospital laboratory and predict that by 2008 up to 45% of testing will be either near patient or self-testing [10]. Revenue from the self-testing market in the US doubled from $1.19 billion in 1994 to $2.34 billion in 2000 and is anticipated to continue to grow. Long waits to see a GP and an increasingly health-conscious population are among the factors thought to contribute to the increased sales [8]. Some areas are growing fast, particularly the use of rapid manual or self-test kits [11].

Reasons for using self-tests may include: tests not being available from a doctor (e.g. PSA tests to young men); convenience; privacy; not wanting to bother doctors or use NHS resources; dissatisfaction with, or mistrust of doctors and/or medicine. In the UK, NHS Direct and drop-in health centres aim to increase access to health services and health information [12]. Such initiatives, together with interventions aiming to redefine patients as consumers, the increased availability of over the counter medication, and funding pressures for health services, have encouraged the development of a self-care culture. People are taking more responsibility for their own health and adopting more consumerist attitudes to health care [13-15]. Self-testing may be part of this. Also, such test results may empower people within a consultation with their doctor, as has been seen in the use of Internet resources [16].

Self-testing is marketed as a valuable way of alerting people to serious health problems so they can seek medical help [17]. Traditionally diagnosis, decision-making and definitions of illness occur within a health service [18]. Having test results in isolation (e.g. slightly raised PSA levels) without an assessment of signs and symptoms may result in inappropriate labelling as a 'patient' [19] and increase perceived risk. Self-testing may have beneficial psychological effects (true negatives), but it could also impact on the delivery of population based screening programmes, increase demands on health care services (investigation and treatment of positive tests), lead to false re-assurance (false negative tests) or raised anxiety, and alter health behaviour/perceptions of risk. There is some evidence that GPs feel that they are increasingly consulted by the 'worried well', that their prescribing behaviour is affected by patient demand, and that they are pressured by their role as gatekeepers to specialist services [20-22], and increased self-testing may exacerbate this situation.

No systematic reviews relating to self-testing generally or self-testing for cancer have been identified. The only UK surveys addressing the issue of self-testing state that 18.3% and 24.9% of people reported that they would prefer self-testing to testing by a doctor and a pharmacist respectively [23], and that 32% of people had bought a self-test kit (although this did include pregnancy tests) [24]. Although other work is ongoing [25], there is currently a lack of published research examining the impact of self-testing on individuals or on the healthcare system. The literature that does exist has limitations; it tends to concentrate on efficacy and reliability [26,27], has been carried out in different health cultures in the US or Europe [28], or is based on opinion only without empirical data [29]. There has been research in areas which may be relevant to self-testing for cancer, for example attendance for routine screening, self examination and use of over the counter medication [30-33]. Such behaviours have been associated with socio-economic status, age, gender, ethnicity, and level of trust of the medical profession [32,34,35]. The relevance of these factors to self-testing for cancer may depend on whether self-testing is used to inform choice and complement standard care, or to challenge standard care.

A variety of cancer related self-tests are available to buy by members of the UK public, including tests for faecal occult blood [36], prostate specific antigen [37], haematuria [38], serum α-Fetoprotein and serum carcinoembryonic antigen (CEA) [39], and breast cancer self-examination kits [40]. Cancer related self-tests are not only widely available but also relatively inexpensive, costs range from less than £1 for an FOB test to £25 for devices to feel for breast irregularities.

Advertising directly to the public for genetic tests for the familial breast cancer genes BRCA1 and BRCA2 began in the USA in 2001 [41]. Advertising cancer genetic services increases demand for products that are likely to be of little benefit outside high-risk families [42]. A number of companies have promoted the idea of "predictive medicine" (using genetic tests to predict the chances that someone will get serious illnesses like cancer), and then offering either lifestyle advice or medication [43,44].

Health Which (December 2002), the press (Guardian November 4 2003 and February 6 2004) and BACUP [45] have advertised the availability of cancer self-test kits from high street chemists. Should Internet sales be shown to be profitable, a wider range of cancer self-test kits is likely to become available from pharmacies. The possible range of new products related to the prevention, diagnosis and treatment of cancer is wide, and potential new developments include a saliva test for breast cancer [46], bladder cancer home tests (currently prescription only) [47], ultraviolet monitors to avoid skin cancer [48], and kits for testing your response to alternative cancer treatments [49].

Cancer related self-testing may develop to include tests for the early diagnosis of cancers at more sites, the genetic determinants of disease [50] and drug effectiveness [51]. The characteristics of those who participate in population based screening and the impact of screening programmes (i.e. costs and benefits to the NHS and participants) have received considerable attention [52]. Almost nothing is known about the extent to which people screen themselves for cancer or why they do this. Self-tests for cancer could alter perceptions of risk and health behaviour, cause psychological morbidity and have a significant impact on the demand for healthcare. Furthermore, they may impact on the cost-effectiveness of population-based screening. It is essential that we gain an understanding of the frequency of self-testing for cancer, characteristics of users and the effects of test results on both users and the health service. It is important that we obtain this information before self-testing for cancer becomes more widely available, to be able to determine the potential impact on both the public and health services.

### Study aims

To estimate the prevalence of cancer-related self-testing use and compare characteristics of users, non-users and potential users of self-tests for cancer.

## Methods

### Study design: community based survey

#### Study population

All adults, over the age of 18, selected from the lists of participating general practices in the West Midlands. The only exclusions will be people who the GP feels it would be inappropriate to send a questionnaire.

#### Recruitment of practices

three to five practices, stratified by Townsend score (census based indicator of deprivation) will be recruited. Lists of eligible persons will be generated from practice registers. These lists will be scrutinised by general practitioners, who will remove all patients where it is deemed inappropriate for a questionnaire to be sent, for example because of recent bereavement, terminal illness or unable to give informed consent.

#### Methods of data collection

Reply-paid postal questionnaire survey of 5000 people selected from the lists of participating practices. Freepost envelopes will be included and non-responders will receive one reminder [53]. This short questionnaire will collect socio-demographic information and basic data regarding previous and potential future use of self-test kits. To minimise response bias, we have incorporated questions relating to self-testing for a range of conditions, rather than just cancer.

### Justification of sample size

Sixty percent of the population have been reported to use self-test kits [8], but the proportion that has used a self-test kit for cancer is unknown. Conservatively assuming that 1% of people have used a cancer-related self-test and a response rate of 47%, mailing 5000 people will allow estimation of the prevalence of the use of self-tests with +-4% precision and 95% confidence [54].

Based on a response rate of 40%, which is less than other large prevalence surveys [55], the questionnaire will be sent to 10500 people. Assuming an average list size of 4500 people, 75% of whom are 18 years or older [56], and 5% of whom meet the exclusion criteria, it would be sufficient to recruit two general practices, but up to five will be recruited to increase generalisability.

### Methods of data analysis

The data will be used to scope the extent and patterns of current cancer-related self-test use and produce profiles of the people who use or would use self-testing for cancer. Participants will be classified according to their use of self-test kits related to cancer. Estimates of the prevalence of the use of self-testing for cancer will be determined after appropriate standardisation to the England and Wales population.

The characteristics of those who have used a self-test kit will be estimated by discriminant analysis. This multivariate technique classifies individuals to known groups on the basis of known information on the individual (e.g. age, gender, Townsend score). The characteristics of patients who have accessed self-testing will be compared with those who have not. The model will aim to identify the predictors of self-test kit use. Logistic regression analysis will be utilised.

### Bias and confounding

The questionnaire will be piloted to ensure readability, comprehension, and acceptability. Participation comprises the completion of a short postal questionnaire. We have kept demands on participants to a minimum to maximise compliance and minimise selection bias.

Given the study design (i.e. unsolicited requests for information), it is inevitable that there will be a significant minority of individuals in the sample who do not respond. In addition, it seems likely that those who do respond will be different in important respects to those who do not. Estimates of response rates by age, sex and deprivation score will be made and standardised prevalence rates will be calculated to overcome some of the potential bias.

#### Ethical approval

This study has been approved by Solihull Local Research Ethics Committee, reference 05/Q2706/13.

## Discussion

Cancer related self-tests, currently available from pharmacies or over the Internet, include FOB kits (related to bowel cancer), PSA tests (related to prostate cancer), breast cancer kits (self-examination guide) and haematuria tests (related to urinary tract cancers) [57]. The range of available tests is likely to increase in the near future. The effect of such an escalation in self-testing for cancer is unknown but may be considerable: it may affect the delivery of population based screening programmes; empower patients or cause unnecessary anxiety; reduce costs on existing healthcare services or increase demand to investigate patients with positive test results. It is important that more is known about the characteristics of those who are using self-tests if we are to determine the potential impact on health services and the public.

## Abbreviations

PSA = prostate specific antigen, FOB = faecal occult blood.

## Competing interests

The author(s) declare that they have no competing interests.

## Authors' contributions

All the authors contributed to the design of the study. Sue Wilson drafted the study protocol with input from all authors. All of the authors have read and approved the final draft.

## Pre-publication history

The pre-publication history for this paper can be accessed here:


